# Effects of Processing and Storage on *Pediococcus pentosaceus* SB83 in Vaginal Formulations: Lyophilized Powder and Tablets

**DOI:** 10.1155/2013/680767

**Published:** 2013-06-17

**Authors:** Sandra Borges, Paulo Costa, Joana Silva, Paula Teixeira

**Affiliations:** ^1^Centro de Biotecnologia e Química Fina (CBQF), Laboratório Associado, Escola Superior de Biotecnologia, Universidade Católica Portuguesa/Porto, Rua Dr. António Bernardino Almeida, 4200-072 Porto, Portugal; ^2^Departamento de Ciências do Medicamento, Laboratório de Tecnologia Farmacêutica, Faculdade de Farmácia, Universidade do Porto, Rua de Jorge Viterbo Ferreira 228, 4050-313 Porto, Portugal

## Abstract

Vaginal probiotics have an important role in preventing the colonization of the vagina by pathogens. This study aimed to investigate different formulations with *Pediococcus pentosaceus* SB83 (lyophilized powder and tablets with and without retarding polymer) in order to verify its stability and antilisterial activity after manufacture and during storage. The bacteriocinogenic activity of *P. pentosaceus* SB83 against *Listeria monocytogenes* was evaluated in simulated vaginal fluid. Suspension of *Pediococcus pentosaceus* SB83 reduced the pathogen only after 2 h and the lyophilized bacteria after 24 h of contact, and, in the tablets, *P. pentosaceus* SB83 lost the antimicrobial activity. The pH of simulated vaginal fluid decreased for all the tested conditions. As lyophilized powder demonstrated better results concerning antimicrobial activity, this formulation was selected to evaluate the antilisterial activity during the 12 months of storage. During storage at room temperature, lyophilized bacteria totally inhibited the pathogen only until one month of storage. At 4°C, *P. pentosaceus* SB83 showed antimicrobial activity during all the time of storage investigated. Therefore, the better formulation of *P. pentosaceus* SB83 is the lyophilized powder stored at 4°C, which may be administered intravaginally as a washing solution.

## 1. Introduction

The dominant microorganisms in vaginal microbiota are lactobacilli, with a concentration of 10^7^ to 10^8^ CFU/mL of vaginal fluid in healthy premenopausal women [1, 2]. However, other lactic acid bacteria (LAB) genera have been found in the vaginal tract, such as *Pediococcus* spp., *Weisella *spp., *Streptococcus* spp., and *Leuconostoc *spp. [3]. Moreover, it is acknowledged that the composition of the microbiota can vary from day to day, even in women without an indication of infections [4].

The normal vaginal pH of a healthy woman is acidic (4–4.5), with variations from 6.6 (+/−0.3) to 4.2 (+/−0.2) between day 2 and day 14 of the menstrual cycle [5], although vaginal pH can rise with the diminishing of LAB existing in the vaginal microbiota, since glucose is not converted to lactic acid. High pH values promote growth of pathogenic organisms, particularly, colonization by enteric bacteria [2].

The microorganisms that are normally present in the vaginal environment play a major role in preventing illnesses of the host, including bacterial vaginosis (BV), urinary tract infections (UTI), yeast vaginitis, and sexually transmitted diseases including human immunodeficiency virus (HIV) [6]. The vaginal LAB are also able to reduce the incidence of ascending infections of the uterus and the subsequent production of proinflammatory molecules [7]. During pregnancy, several genital microorganisms such as *Escherichia coli*, *Listeria monocytogenes,* and viridans streptococci may be involved in chorioamnionitis [8]. 

Antimicrobial treatment of urogenital infections is not always effective, and problems remain due to bacterial and yeast resistance. To minimize recurrent infections, as well as side effects, it is important to develop and produce alternative treatments or drugs [9].

Due to the beneficial characteristics of LAB, there has been a growing interest in the potential use of LAB as probiotics for maintaining normal urogenital health [10]. The World Health Organization and the Food and Agriculture Organization of the United Nations have defined probiotics as “live microorganisms, which, when administered in adequate amounts, confer a health benefit on the host” [11].

A probiotic may act indirectly through treating and preventing recurrent BV or directly by secreting substances (e.g., hydrogen peroxide, bacteriocins, and lactic acid) that inhibit pathogens [12].

Vaginal dosage forms available include creams, gels, tablets, capsules, pessaries, foams, ointments, films, tampons, rings, and douches. While the majority of vaginal drugs so far have been in the form of gels, there is a growing interest in alternative dosage forms such as rings, tablets, and films. The majority of issues with product development and scaleup are similar to other pharmaceutical products, although there are some unique challenges because of the site of delivery, prophylactic nature of product application, and diversity in sex and hygiene practices across the developing world [13].

The purpose of this study was to develop a vaginal formulation containing viable cells of a LAB isolate which can be used by women to prevent vaginal colonization of microorganisms (e.g., *L. monocytogenes*) that may be harmful to the fetus/neonate. In this study the probiotic properties (survival and antagonistic activity) of formulations were evaluated after manufacture and during long-term storage of the product at different temperatures.

## 2. Material and Methods 

### 2.1. Microorganisms and Culture Conditions

*Pediococcus pentosaceus* SB83 (Escola Superior de Biotecnologia Culture Collection) was selected because previous studies demonstrated that this strain is able to inhibit the growth of clinical isolates of *L. monocytogenes* (serotype 1/2a, 1/2b and 4b) by production of a bacteriocin [14, 15]. This strain has been shown to survive in simulated vaginal fluid at normal vaginal pH (pH of 4.2), and *P. pentosaceus* SB83 does not produce virulence factors such as gelatinase, lipase and DNase, and hemolytic activity, nor does it possess virulence genes (genes *esp*, *agg*, *gel*E, *efaAfm*, *efaAfs*,* cylA*, *cylB,* and c*ylM*) [15]. 

*Pediococcus pentosaceus* SB83 was grown on de Man, Rogosa and Sharpe (MRS) agar (Lab M, Bury, United Kingdom) and was subcultured twice in MRS broth (Lab M) at 37°C for 24 h before use in all tests. 

The pathogenic bacteria, *L. monocytogenes* 2092 4b (isolated from blood; Escola Superior de Biotecnologia Culture Collection), was grown on Tryptone Soya agar with Yeast Extract 0.6% (w/v) (TSA-YE, Lab M) and subcultured in Tryptone Soya broth with Yeast Extract (TSB-YE, Lab M) at 37°C for 24 h.

All strains were stored at −80°C in the presence of 30% (v/v) glycerol (Panreac, Barcelona, Spain).

### 2.2. Preparation of Freeze-Dried Cells

After growth of *P. pentosaceus* SB83 in MRS broth, cultures were harvested by centrifugation at 8877 ×g for 10 min at 4°C and washed twice in sterile Ringer's solution (Oxoid, Hampshire, United Kingdom). The harvested cells were then suspended to the original volume in 11% (w/v) of skim milk (Oxoid). The cultures were frozen at −80°C for 24 h and subsequently freeze-dried (Martin Christ, Osteradam Harz, Germany) for 2-3 days.

### 2.3. Preparation of Vaginal Tablets

The formulations of vaginal tablets constituted different excipients: 3.3% talc (Acofarma, Barcelona, Spain), 3.0% magnesium stearate (J.M.V. Pereira, Portugal), 0.2% colloidal silicon dioxide (Acofarma), 7.5% hydroxypropyl methylcelluloses (HPMC, Acofarma) as retarding polymer, and 86.0% of lyophilized bacteria. Simultaneously, tablets without the retarding polymer were also prepared.

 The HPMC are known to show bioadhesive properties, but they are also able to hydrate and gel very slowly due to their high molecular weights and viscosities; for this reason, the tablet dissolves over a longer time and provides a prolonged release of the active ingredient embedded in the polymeric matrix [16]. The powder contained an amount of magnesium stearate as lubricant and talc as antiadherent [17].

All compounds were mixed in a Turbula apparatus for 15 min. Then, tablets were prepared by direct compression using a single-punch tablet press (Specac Ltd., Kent, United Kingdom). A quantity of the mixture (approximately 600 mg) was filled into a die of 11 mm diameter and under a pressure of 9.8 kN, and tablets with a plane surface were formed.

The possible adverse effects of pressing on bacterial viability were investigated by comparing the viability of cells in tablets with those in formulated powders.

### 2.4. Release Study of Vaginal Tablets

The determination of the release of *P. pentosaceus* SB83 from vaginal tablets was done using simulated vaginal fluid (SVF). Simulated vaginal fluid was prepared according to Owen and Katz [18]: 3.5 g/L NaCl (Panreac); 1.4 g/L KOH (Pronalab, Lisbon, Portugal); 0.2 g/L Ca(OH)_2_ (Merck, Darmstadt, Germany); 0.02 g/L bovine serum albumin (Sigma-Aldrich, Steinheim, Germany); 2.0 g/L lactic acid (Fluka, Steinheim, Germany); 1.0 g/L acetic acid (Panreac); 0.2 g/L glycerol; 0.4 g/L urea (Sigma-Aldrich); 5.0 g/L glucose (Pronalab). Once these compounds were combined in solution, the mixture was adjusted to a pH of 4.2, 5.5, and 6.5 using HCl (Pronalab) or NaOH (Pronalab). These pH values were used to reproduce the vaginal pH in normal conditions (pH 4.2) and in the case of infection (pH 5.5 and 6.5). As control, Ringer's solution was used.

Tablets with and without retarding polymer were placed in ten millilitres of each solution at 37°C, since the daily production of vaginal fluid ranges between 1.89–11.00 mL/day [18]. At appropriate time intervals, aliquots were withdrawn for further enumeration of viable bacteria. Two independent replicates of these assays were performed. The enumeration of *P. pentosaceus* SB83 was performed by serial 10-fold dilutions in Ringer's solution, and 20 *μ*L was spread-plated in duplicate on MRS agar. Colony forming units per gram (CFU/g) was determined after 48 h of incubation at 37°C. 

### 2.5. Viability of *Pediococcus pentosaceus* SB83 in Tablets and Lyophilized Powder

Freeze-dried powder and pharmaceutical preparations (freeze-dried powder with excipients and tablets) were stored in plastic containers at room temperature and at 4°C for 12 months. At several time intervals (0, 1, 3, 6, 9, and 12 months), the viability of *P. pentosaceus* SB83 was determined by plate method using MRS agar medium (described above). The detection limit of the enumeration technique was 1.4 log CFU/g.

### 2.6. Evaluation of Antimicrobial Activity of *Pediococcus pentosaceus* SB83 in Simulated Vaginal Fluid

*Pediococcus pentosaceus* SB83 produce a bacteriocin against *L. monocytogenes* that is resistant to the components and pH of vaginal fluid [14].

The bacterial suspension, lyophilized powder, and tablets were used in these experiments to evaluate the antimicrobial activity of* P. pentosaceus *SB83 in SVF. To obtain the bacterial suspension, the microorganism was subcultured twice (24 h at 37°C) in 10 mL MRS broth, using a 1% v/v inoculum. Then cultures were harvested by centrifugation at 8877 ×g for 10 min at 4°C, washed twice in sterile Ringer's solution, and the pellet of bacteria was used. The lyophilized powder and tablets were prepared as described above.

Simulated vaginal fluid at pH of 6.5 was inoculated with an overnight culture of *L. monocytogenes* 2092 4b suspended in Ringer's solution with a final concentration of 10^5^ CFU/mL. Then, *P. pentosaceus* SB83 (bacterial suspension, lyophilized powder, or tablet) was added.

During 24 h, aliquots were withdrawn for further enumeration (*P. pentosaceus* SB83 and *L. monocytogenes* 2092 4b) and for pH measurement. The enumeration of *P. pentosaceus* SB83 was performed on MRS agar and the enumeration of *L. monocytogenes *on PALCAM agar (Merck), after 48 h incubation at 37°C. 

The experimental conditions were (1) SVF inoculated with *P. pentosaceus* SB83 (bacterial suspension, lyophilized powder, tablet with retarding polymer, and tablet without retarding polymer) and *L. monocytogenes* 2092 4b, (2) SVF with 1 mg/mL of trypsin (Sigma-Aldrich) and inoculated with *P. pentosaceus* SB83 and *L. monocytogenes* 2092 4b, (3) SVF inoculated with a nonbacteriocinogenic LAB strain ESB67 (Escola Superior de Biotecnologia Culture Collection) and *L. monocytogenes* 2092 4b, (4) SVF inoculated with *P. pentosaceus* SB83, and (5) SVF inoculated with *L. monocytogenes* 2092 4b. Each trial was performed in duplicate.

After this experiment, the formulation of *P. pentosaceus *SB83 that demonstrated the better results of antilisterial activity was selected; the formulation was then stored at room temperature and 4°C for 12 months. At 1, 3, 6, 9, and 12 months, the evaluation of antimicrobial activity of *P. pentosaceus *SB83 was performed as previously described.

### 2.7. Statistical Analysis

An analysis of variance (ANOVA) was carried out to assess (1) the effect of pH in the release of *P. pentosaceus* SB83 in tablets with and without retarding polymer, (2) the effect of time and temperature of storage on viability of *P. pentosaceus *SB83 in tablets and lyophilized powder, (3) the effect of different forms of *P. pentosaceus *SB83 (bacterial suspension, lyophilized powder, and tablets) for antilisterial activity and reduction of pH of SVF, and (4) the effect of storage on antilisterial activity and reduction of pH of SVF. All calculations were carried out using the software Kaleidagraph (version 4.04, Synergy Software, Reading, USA).

## 3. Results

### 3.1. Enumeration of *Pediococcus pentosaceus* SB83 in Lyophilized Powder and Tablets

The number of *P. pentosaceus* SB83 in lyophilized powder and in tablets (with and without retarding polymer) was 10^10^ CFU/g.

### 3.2. Release Study of Vaginal Tablets

**Figure 1:**
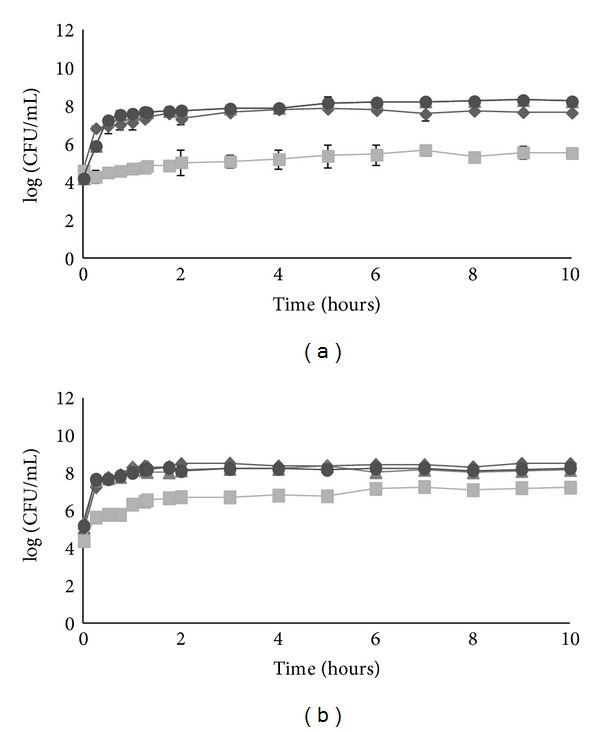
Release of *Pediococcus pentosaceus* SB83 from (a) tablets with retarding polymer and (b) tablets without retarding polymer. The release of bacteria was tested in SVF at pH = 4.2 (■); pH = 5.5 (▲); pH = 6.5 (*⬤*) and in Ringer's solution (*◆*). All points are means ± standard deviations.

The release of *P. pentosaceus* SB83 from tablets with and without retarding polymer is shown in Figure 1.

In tablets with retarding polymer, *P. pentosaceus* SB83 was not completely released in all media tested. In SVF at pH 4.2 a lower release was observed (approximately 5.0 log CFU/g) than the release in the other media (SVF at pH 5.5, SVF at pH 6.5 and Ringer's solution) that was approximately 8.0 log CFU/g.

In tablets without retarding polymer, *P. pentosaceus* SB83 was completely released after 48 h in SVF at pH 5.5, SVF at pH 6.5, and Ringer's solution. The release of bacteria was lower in SVF at pH 4.2, with values being approximately 8.0 log CFU/g. 

Therefore, in both tablets (with and without retarding polymer) the bacteria release characteristics were found to be different when comparing the release in SVF at pH 4.2 with the other media (*P* < 0.0001).

In either of the tablets, there was a large release of bacteria in the early hours of contact.

### 3.3. Viability of *Pediococcus pentosaceus* SB83 in Tablets and Lyophilized Powder

**Figure 2:**
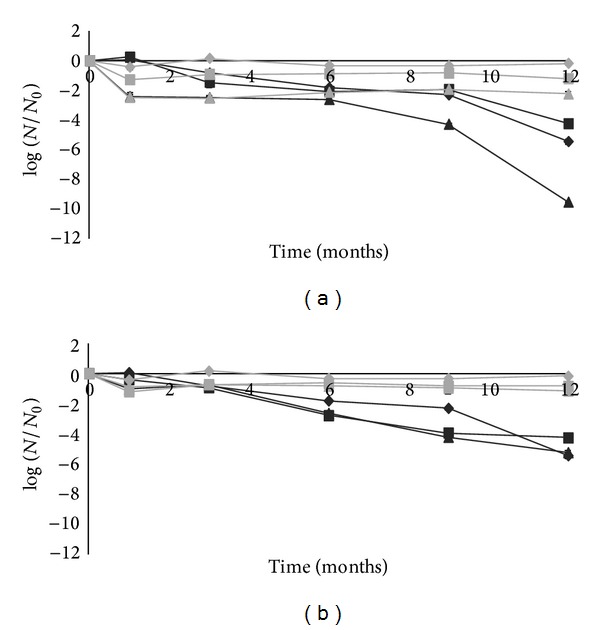
Viability of *Pediococcus pentosaceus* SB83 in preparations with (a) retarding polymer and (b) without retarding polymer during storage. The viability was examined in freeze-dried powder (*◆*), freeze-dried powder with excipients (■), and tablets (▲) at room temperature (black lines) and at 4°C (grey lines). All points are means ± standard deviations.

Figure 2 shows the viability of *P. pentosaceus* SB83 in lyophilized powder and pharmaceutical formulations (lyophilized powder with excipients and tablets) during storage at room temperature and 4°C.

At room temperature, in conditions with retarding polymer (Figure 2(a)), the survival was similar during 9 months of storage (*P* = 0.09). However, after 12 months of storage, the tablets showed a greater decrease of viable cells (reduction of 9.5 log CFU/g) than that of lyophilized powder and lyophilized powder with excipients. In conditions without retarding polymer (Figure 2(b)), no significant differences were found between lyophilized powder, lyophilized powder with excipients, and tablets (*P* = 0.18) during storage.

At 4°C, in both conditions (with and without retarding polymer) a higher survival of *P. pentosaceus *SB83 was recorded during storage than at room temperature (*P* < 0.05).

### 3.4. Evaluation of Antimicrobial Activity of *Pediococcus pentosaceus* SB83 in Simulated Vaginal Fluid

**Figure 3:**
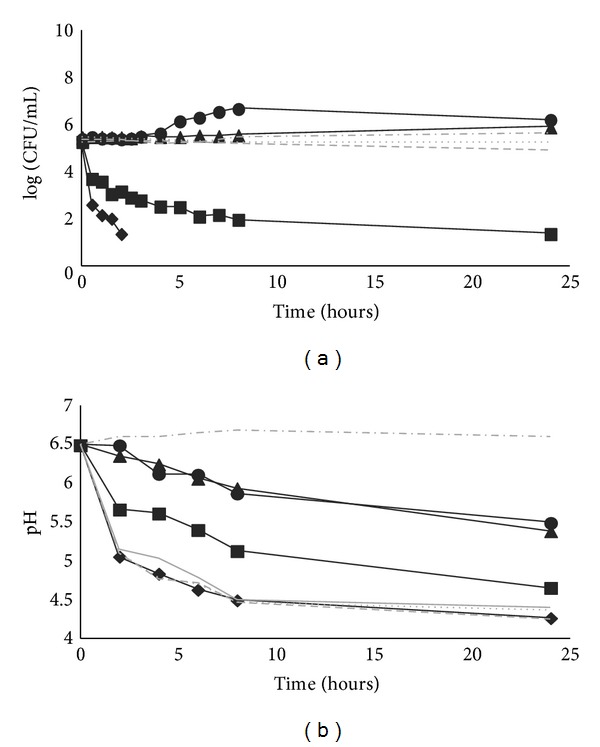
Antimicrobial activity of *Pediococcus pentosaceus* SB83 against *Listeria monocytogenes* 2092 4b in simulated vaginal fluid at pH = 6.5. (a) Counts of viable cells of *Listeria monocytogenes* 2092 4b and (b) pH of simulated vaginal fluid during 24 h of contact, are represented. The tested conditions were SVF inoculated with bacterial suspension of *P. pentosaceus* SB83 and *L. monocytogenes* 2092 4b (*◆*); lyophilized of *P. pentosaceus* SB83 and *L. monocytogenes* 2092 4b (■); tablet of *P. pentosaceus* SB83 with retarding polymer and *L. monocytogenes* 2092 4b (▲), tablet of *P. pentosaceus* SB83 without retarding polymer and *L. monocytogenes* 2092 4b (*⬤*). Controls were: SVF with 1 mg/mL of trypsin and inoculated with *P. pentosaceus* SB83 and *L. monocytogenes* 2092 4b (***⋯***), SVF inoculated with a non-bacteriocinogenic LAB strain ESB67 and *L. monocytogenes* 2092 4b (**– –**), and SVF inoculated with *P. pentosaceus* SB83 (**—**), SVF inoculated with *L. monocytogenes* 2092 4b (**– ·**). All points are means ± standard deviations.

Figure 3 shows the antimicrobial activity *of P. pentosaceus* SB83 (in bacterial suspension, lyophilized powder, and tablets with and without retarding polymer) against* L. monocytogenes* 2092 4b, in SVF at pH = 6.5.

The bacterial suspension of *P. pentosaceus* SB83 reduced *L. monocytogenes* 2092 4b below the detection limit after only 2 h. The lyophilized bacteria inhibited* Listeria* population mainly over 8 h of contact; however, total inhibition (up to the limit of detection) was recorded after 24 h. The tablets did not reduce *L. monocytogenes* 2092 4b during 24 h of experiment (*P* = 0.11) (Figure 3(a)).

The viable counts of *P. pentosaceus* SB83 were maintained throughout the 24 h in all conditions tested (data not shown).

All conditions tested, demonstrated the ability to decrease the pH of SVF (Figure 3(b)). However, the highest reduction of pH was observed for bacterial suspension, followed by the lyophilized cells. The lowest reduction of pH was observed when tablets formulations were used. No significant difference was achieved for tablets with and without retarding polymer (*P* = 0.94). 

**Figure 4:**
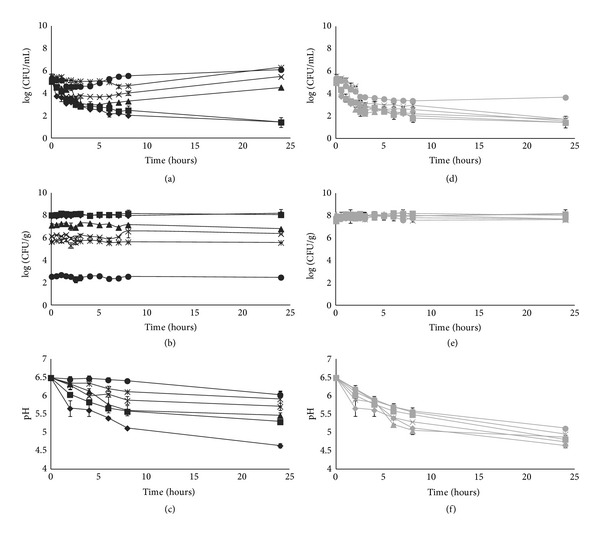
Antimicrobial activity of lyophilized of *Pediococcus pentosaceus* SB83 against *Listeria monocytogenes* 2092 4b in simulated vaginal fluid at pH = 6.5 during storage at room temperature (black lines) and at 4°C (grey lines). ((a) and (d)) Counts of viable cells of *Listeria monocytogenes* 2092 4b; ((b) and (e)) counts of viable cells of *Pediococcus pentosaceus* SB83; ((c) and (f)) pH of simulated vaginal fluid overtime are represented. These parameters were evaluated at storage time of 0 (*◆*), 1 (■), 3 (▲), 6 (**×**), 9 (*җ*), and 12 (*⬤*) months. All points are means ± standard deviations.

After obtaining these results, lyophilized bacteria were selected to evaluate the antilisterial activity during storage at room temperature and at 4°C for 12 months (Figure 4).

The lyophilized bacteria, when stored at room temperature, totally reduced *L. monocytogenes* 2092 4b (up to the limit of detection) until one month of storage. After 3 months, a decrease in antimicrobial capability was observed. From 3 to 12 months of storage, there was a similar behaviour (*P* > 0.05), and the pathogen was reduced in the early hours of contact with the LAB isolate, though there was an increase of *L. monocytogenes* 2092 4b after 24 h (Figure 4(a)).

When storage was performed at 4°C, the antimicrobial activity of *P. pentosaceus* SB83 was maintained during storage (*P* = 0.66), showing only a slight decrease in antimicrobial potential after 12 months (Figure 4(d)).

As previously observed, lyophilized *P. pentosaceus *SB83 were more stable at 4°C than room temperature (Figures 4(b) and 4(e)). 

During storage of the lyophilized bacteria at room temperature, the ability of *P. *pentosaceus SB83 to reduce the pH of SVF decreased over time (*P* < 0.001) (Figure 4(c)). In contrast, during storage at 4°C the ability to reduce the pH of SVF throughout the time of storage was observed. This capacity only decreased slightly with time (*P* = 0.66; Figure 4(f)).

## 4. Discussion

The intravaginal route is normally used for the administration of drugs such as antimicrobials, labour-inducing agents, spermicidal agents, prostaglandins, and steroids [19]. Accordingly, the vaginal route can be used to prevent vaginal colonization by pathogenic microorganisms in pregnant women, and thereby is a method of protection of the fetus/neonate. 

Based on this principle, different pharmaceutical formulations *with P. pentosaceus* SB83, a bacteriocin-producing LAB [14], were performed for subsequent vaginal administration. 

In this study freeze-dried cells of *P. pentosaceus* SB83 were used, containing 10^10^ CFU/g, which remained after the production of tablets, so tabletting had no adverse effect on the viability of bacteria. Generally, the quantity used for vaginal probiotics is between 10^8^ and 10^10^ CFU in the product [16, 20–22]. 

The normal vaginal pH in premenopausal women is acidic; however, vaginal pH can be increased due to a depletion of vaginal lactobacilli [23], menstruation, unprotected sexual intercourse with the deposition of semen [24], and vaginal medications. Therefore, we tested the release of *P. pentosaceus *SB83 in SVF at different values of pH (4.2, 5.5, and 6.5). The pH influenced the release of bacteria from tablets. Tablets with retarding polymer as well as tablets without retarding polymer released fewer cells of *P. pentosaceus* SB83 at pH 4.2.

The presence of retarding polymer affected the total numbers of bacteria released. The polymer used (HPMC) retained *P. pentosaceus *SB83 inside the tablets, releasing a maximum of 10^8^ CFU/g. However, this release occurred in the early hours of contact with SVF, such as in tablets without the retarding polymer. Fazeli et al. [25] performed an assay with tablets with HPMC and concluded that a high initial bacterial load release occurs during the first 15 min in deionized sterile water; however, the tablets showed a continuous bacterial release during 5.75 h. 

The tablets and lyophilized powder were stored during 12 months in order to determine their stability. There was an effect of temperature on viability of cells during storage; *P. pentosaceus* SB83 showed a higher survival rate at 4°C than at room temperature. According to other studies, freeze-dried bacteria are more stable at low temperatures than at room temperature [17, 26]. It was reported that milk promotes survival at low temperature by stabilizing the cell membrane constituents and forming a protective coating on the cell wall proteins (see review [27]).

At 4°C the viability of *P. pentosaceus* SB83 was maintained at a high level in all formulations tested for at least 12 months. Fazeli et al. [25] also demonstrated that slow-release tablets were stable at 4°C during 6 months of storage. Maggi et al. [16] tested the viability of 10 lactobacilli strains in freeze-dried powder and tablets and have shown that the stability depends on the bacterial strain and also on the polymer used as a retarding compound. In their study, four strains of lactobacilli maintained a high viability for 18 months at 4°C.

While observing an elevated survival of bacteria during storage, it is important to confirm that the antimicrobial characteristics are also maintained. In previous studies, our group characterized the bacteriocin produced by *P. pentosaceus* SB83 in MRS medium [14] and tested the antimicrobial activity of *P. pentosaceus* SB83 after exposure to SVF at pH 4.2 after 24 h [15]. However, it is relevant to evaluate the production and effectiveness of bacteriocin production by the LAB after storage, in SVF, in case of vaginal colonization by pathogens (elevated pH). The pathogen selected to assess these parameters was *L. monocytogenes*. This bacterium can be acquired via vertical transmission from mother to fetus/neonate through the placenta or birth canal [28, 29] and can lead to preterm labour, chorioamnionitis, spontaneous abortion, stillbirth, and neonatal infection [30].

 In experiments to determine the inhibitory activity of stored cells of *P. pentosaceus* SB83, SVF at pH of 6.5 was used because it was demonstrated previously that clinical strains of *L. monocytogenes *are inhibited by the normal vaginal pH for 48 h but may proliferate when the pH increases [31].

The bacterial suspension of *P. pentosaceus* SB83 reduced *L. monocytogenes* 2092 4b below the detection limit after only 2 h (reduction of approximately 4.0 log). The pH of SVF was decreased to normal vaginal pH (4.2) in 24 h.

The lyophilized bacteria showed a total inhibition of the pathogen after 24 h of contact. The pH of SVF decreased to values of 4.5 in 24 h.

The tablets did not reduce counts of *L. monocytogenes* 2092 4b, although the bacteria in the tablets were able to decrease the pH to values of 5.5 in 24 h. These results showed that the activity of the bacteriocin SB83 was affected by the excipients used in production of tablets, probably by its adsorption to the excipients. It has been reported that some compounds commonly used as pharmaceutical excipients and/or starter culture cryoprotectants can alter or completely inhibit the activity of bacteriocins [32]. The reduction in pH was rather less by cells in tablets than by lyophilized cells; this also suggests that the tabletted cells are rather less metabolically active than lyophilized cells.

In the controls used (SVF with 1 mg/mL of trypsin and inoculated with *P. pentosaceus* SB83 and *L. monocytogenes* 2092 4b; SVF inoculated with a nonbacteriocinogenic LAB strain ESB67 and *L. monocytogenes* 2092 4b), there was no inhibition of pathogen, while there was a decrease of pH. This suggests that the antimicrobial activity observed in bacterial suspension and lyophilized in SVF was due to the production of bacteriocin SB83. 

The studies performed on the production of bacteriocins in the vaginal environment are very few. According to the study by Tomás and Nader-Macías [33], *Lactobacillus salivarius* CRL 1328 was able to grow in SVF at 4.25, but that bacteriocin production was minimal, only 40 AU/mL.

The lyophilized powder demonstrated antimicrobial activity; therefore, this formulation was selected to evaluate the antilisterial activity during the 12 months of storage. In pharmaceutical products it is important to verify the stability of the bioactive compounds and the maintenance of the biological activity during long periods of storage.

When stored at room temperature, lyophilized cells inhibited totally the pathogen (up to the limit of detection) up to one month of storage. After this time, there was a decrease in antimicrobial potential that may be caused by diminishing numbers of viable cells of *P. pentosaceus* SB83 during storage at room temperature. This decrease in the number of cells could also have influenced the reduction of pH of SVF; the production of acid is lower with storage time of *P. pentosaceus* SB83 cells.

When lyophilized cells were stored at 4°C, *P. pentosaceus* SB83 maintained their viability, and consequently the anti-listerial activity and the capacity to produce acid were observed throughout the time of storage. The bacteriocin SB83 remained active during at least 12 months in spite of a decrease that occurred in antimicrobial activity at the twelfth month of storage. 

Zárate and Nader-Macias [32] evaluated the bacteriocinogenic activity of* L. salivarius* CRL 1328 after lyophilization (with different protective substances, such as lactose, skim milk, ascorbic acid, and combinations of them) during storage at 5°C. It was demonstrated that bacteriocin synthesis was not affected by the lyophilization, but a slight decrease of activity was observed during storage in cells lyophilized with lactose and completely abolished in cells lyophilized with ascorbic acid.

Pingitore et al. [34] tested the activity of a lyophilized bacteriocin (salivaricin) during storage at −25°C, 4°C, and 25°C for 18 months. The antimicrobial activity of salivaricin was dependent mainly on temperature and also on the time of storage and protectant used to lyophilize. The stability of salivaricin was higher at temperatures lower than 25°C and decreased during the time of storage.

To our knowledge, this is the first study where the bacteriocin production of lyophilized bacteria was tested against *L. monocytogenes* under the conditions of vaginal fluid and throughout 12 months of storage.

The assays for antimicrobial activity were performed against *L. monocytogenes*; nevertheless bacteriocin SB83 also inhibits the growth of *Enterococcus* spp. [14]; thus, the lyophilized cells may also be used to prevent UTI caused by this pathogen.

*Pediococcus pentosaceus* SB83 showed the ability to reduce the pH of SVF, which is relevant because in addition to inhibiting those pathogens previously mentioned, this LAB has the capability of preventing colonization by other vaginal pathogens by reduction of vaginal pH acid; one study reported the inhibition of group B *Streptococcus* in SVF at pH 4.2 [35]. Moreover, the maintenance of the acidic vaginal pH will allow the proliferation of the vaginal *Lactobacillus* spp. belonging to the normal microbiota of the vaginal tract. Hemalatha et al. [36] demonstrated that a probiotic lactobacilli tablet was found to be better than a pH lowering vaginal tablet in preventing BV in healthy subjects.

## 5. Conclusions

The better formulation of *P. pentosaceus* SB83 is the lyophilized powder which may be administered vaginally as a washing solution. Based on the tests carried out, the product must be stored at 4°C and can be used for a period of time of 9 months. If storage is performed until this time, the characteristics of cells are maintained, that is, the number of viable cells, the production of an active bacteriocin, and the ability to reduce vaginal pH.

Further assays need to be performed, including adhesion to vaginal epithelial cells and clinical trials, to determine the frequency of administration and the effectiveness of *in vivo* of the lyophilized powder of *P. pentosaceus* SB83. Other formulations can be tested using lyophilized bacteria, including tablets with suitable excipients, ovules, and gels.
